# A Novel Antibody-Drug Conjugate (ADC) Delivering a DNA Mono-Alkylating Payload to Chondroitin Sulfate Proteoglycan (CSPG4)-Expressing Melanoma

**DOI:** 10.3390/cancers12041029

**Published:** 2020-04-22

**Authors:** Ricarda M. Hoffmann, Silvia Crescioli, Silvia Mele, Eirini Sachouli, Anthony Cheung, Connie K. Chui, Paolo Andriollo, Paul J. M. Jackson, Katie E. Lacy, James F. Spicer, David E. Thurston, Sophia N. Karagiannis

**Affiliations:** 1 St. John’s Institute of Dermatology, School of Basic & Medical Biosciences, King’s College London, Tower Wing, 9th Floor, Guy’s Hospital, London SE1 9RT, UK; 2 NIHR Biomedical Research Centre at Guy’s and St. Thomas’s Hospitals and King’s College London, King’s College London, London SE1 9RT, UK; 3 Breast Cancer Now Research Unit, School of Cancer & Pharmaceutical Sciences, King’s College London, Guy’s Cancer Centre, London SE1 9RT, UK; 4 Institute of Pharmaceutical Science, School of Cancer and Pharmaceutical Sciences, King’s College London, London SE1 9NH, UK; 5 Femtogenix Ltd, Lawes Open Innovation Hub, Rothamsted Research, West Common, Harpenden, Hertfordshire AL5 2JQ, UK; 6 School of Cancer & Pharmaceutical Sciences, King’s College London, 3rd Floor, Guy’s Hospital, London SE1 9RT, UK

**Keywords:** antibody-drug conjugate (ADC), antibodies, CSPG4, HMW-MAA, NG2, DNA-binding agent, mono-alkylating, sequence-selective, melanoma

## Abstract

Despite emerging targeted and immunotherapy treatments, no monoclonal antibodies or antibody-drug conjugates (ADCs) directly targeting tumor cells are currently approved for melanoma therapy. The tumor-associated antigen chondroitin sulphate proteoglycan 4 (CSPG4), a neural crest glycoprotein over-expressed on 70% of melanomas, contributes to proliferative signaling pathways, but despite highly tumor-selective expression it has not yet been targeted using ADCs. We developed a novel ADC comprising an anti-CSPG4 antibody linked to a DNA minor groove-binding agent belonging to the novel pyrridinobenzodiazepine (PDD) class. Unlike conventional DNA-interactive pyrrolobenzodiazepine (PBD) dimer payloads that cross-link DNA, PDD-based payloads are mono-alkylating agents but have similar efficacy and substantially enhanced tolerability profiles compared to PBD-based cross-linkers. We investigated the anti-tumor activity and safety of the anti-CSPG4-(PDD) ADC in vitro and in human melanoma xenografts. Anti-CSPG4-(PDD) inhibited CSPG4-expressing melanoma cell growth and colony formation and triggered apoptosis in vitro at low nanomolar to picomolar concentrations without off-target Fab-mediated or Fc-mediated toxicity. Anti-CSPG4-(PDD) restricted xenograft growth in vivo at 2 mg/kg doses. One 5 mg/kg injection triggered tumor regression in the absence of overt toxic effects or of acquired residual tumor cell resistance. This anti-CSPG4-(PDD) can deliver a highly cytotoxic DNA mono-alkylating payload to CSPG4-expressing tumors at doses tolerated in vivo.

## 1. Introduction

Antibody-drug conjugates (ADCs) deliver highly cytotoxic payloads directly to cancer cells by linking the payload to a monoclonal antibody that recognizes a tumor antigen [1]. The design of an ADC entails selection of an appropriate cell surface target antigen, and subsequent generation of an antibody capable of internalizing in the cancer cells followed by release of the payload [2]. Ideally, the payload should be able to kill non-dividing as well as dividing cancer cells, and released only at the tumor site, thus sparing healthy tissue from exposure to its toxic effects [3]. Currently, seven ADCs are approved by the regulatory agencies, five of which are for the treatment of hematopoietic tumors [1]. Trastuzumab emtansine (Kadcyla^®^, Genentech, containing the maytansinoid payload DM1) was approved in 2013 [4] and trastuzumab dexrutecan (Enhertu^®^, Daiichi Sankyo/Astra Zeneca) was approved in 2019 for the treatment of HER2-expressing breast cancers, and both provide confidence that ADCs can be designed to treat other solid tumor types [5]. Along with seven ADCs, two immunotoxins, moxetumomab pasudotox (Lumoxiti^®^, Innate Pharma S.A./Astra Zeneca) [6] and tagraxofusp (Elzonris^®^, Stemline Therapeutics, Inc.) [7], have been approved for the treatment of hairy cell leukemia and blastic plasmacytoid dendritic cell neoplasm, respectively. 

Presently, patients with malignant melanoma, the most lethal form of skin cancer, do not yet benefit from specifically-targeted antibody therapeutics and ADCs that directly target melanoma cells. Although monoclonal antibodies recognizing T cell checkpoint ligands and receptors have significantly improved patient outcomes [8,9,10], treatments are often accompanied by high toxicities, and half of patients either do not respond or develop resistance [11,12]. Previous efforts to develop anti-melanoma ADCs include DEDN6526A (Seattle Genetics/Genentech) [13] targeted to the endothelin B receptor (ETBR) and glembatumumab vedotin (Celldex) [14] recognizing the transmembrane glycoprotein NMB (GPNMB), both carrying the antimitotic payload monomethyl auristatin E (MMAE, vedotin). These were not progressed beyond Phase I and Phase II trials, respectively [13,14,15], although other ADCs carrying MMAE payloads (e.g., brentuximab vedotin [Adcetris^®^, Seattle Genetics] [16] and polatuzumab vedotin [Polivy^©^, Genentech] [17]) are approved to treat lymphomas. An ADC designed to target drug-resistant melanomas expressing the receptor tyrosine kinase AXL conjugated to MMAE (AXL-107-MMAE, Genmab) showed cooperative inhibition of melanoma growth when combined with BRAF/MEK inhibitors [18], highlighting the importance of targeting tumor vulnerabilities when designing ADCs. Therefore, there is an incentive to develop an effective ADC for the treatment of melanoma. 

A large proportion (i.e., ~70%) of malignant melanomas express the cell surface glycoprotein melanoma-associated antigen chondroitin sulfate proteoglycan (CSPG4) [19]. While highly-expressed in melanomas, its restricted and low normal tissue distribution may help limit on-target toxicities [20]. A neural crest marker, CSPG4 participates in melanoma pathogenesis and progression through activation of key signaling pathways (e.g., MAPK/ERK1,2) and through the promotion of sustained high-level activating signals required for malignant progression [20,21,22]. Hence, targeting antibodies and ADCs directly to melanoma cells with highly aggressive behavior could improve outcomes for a significant proportion of patients [23].

Clinical results from the approved ADCs and those in clinical development suggest that the anticipated improvement in Therapeutic Index (TI) has not yet been realized [24], in part due to the toxic effects of the first-generation payloads which target DNA (i.e., calicheamicins, PBD dimers) or tubulin (maytansinoids, auristatins) [25]. Tubulin inhibitors (i.e., the auristatins and maytansinoids) only kill proliferating cells, and may lack efficacy in killing non-dividing tumor cells [26,27]. For the PBD dimers, estimates suggest that just one molecule reaching a healthy cell will form a persistent DNA cross-link leading to cell death, but this problem only manifests when the cell attempts to divide [28]. The pyrridinobenzodiazepines (PDDs) are a new class of DNA-interactive ADC payload that form mono-alkylated adducts rather than the cross-linked adducts formed by the PBD dimers. These adducts are easier for healthy cells to repair compared to the PBD dimer adducts, providing less off-target toxicity. Due to their DNA sequence-selectivity, introduced with the help of a molecular modelling design process, PDD-based payloads bind specifically to transcription factor binding sites which may provide another level of selectivity [29]. The PDD payloads are also designed to have minimal hydrophobicity which optimizes conjugation to an antibody [29,30]. Together these properties can lead to highly targeted cytotoxicity towards tumor cells [31].

In this study we have taken advantage of the DNA minor-groove targeted cytotoxicity of the PDDs, and the highly specific targeting ability of antibodies, to provide the means to deliver these toxic PDD agents directly to melanoma cells through recognition of the CSPG4 cell surface antigen over-expressed by a high proportion of aggressive melanoma tumors. Here we describe the in vitro and in vivo anti-tumor activity of a novel ADC based on an engineered human/mouse chimeric anti-CSPG4 antibody and a DNA mono-alkylating PDD payload.

## 2. Results

### 2.1. Anti-CSPG4 Monoclonal Antibody Generation and Evaluation of Internalizing Potential by Tumor Cells

**Figure 1:**
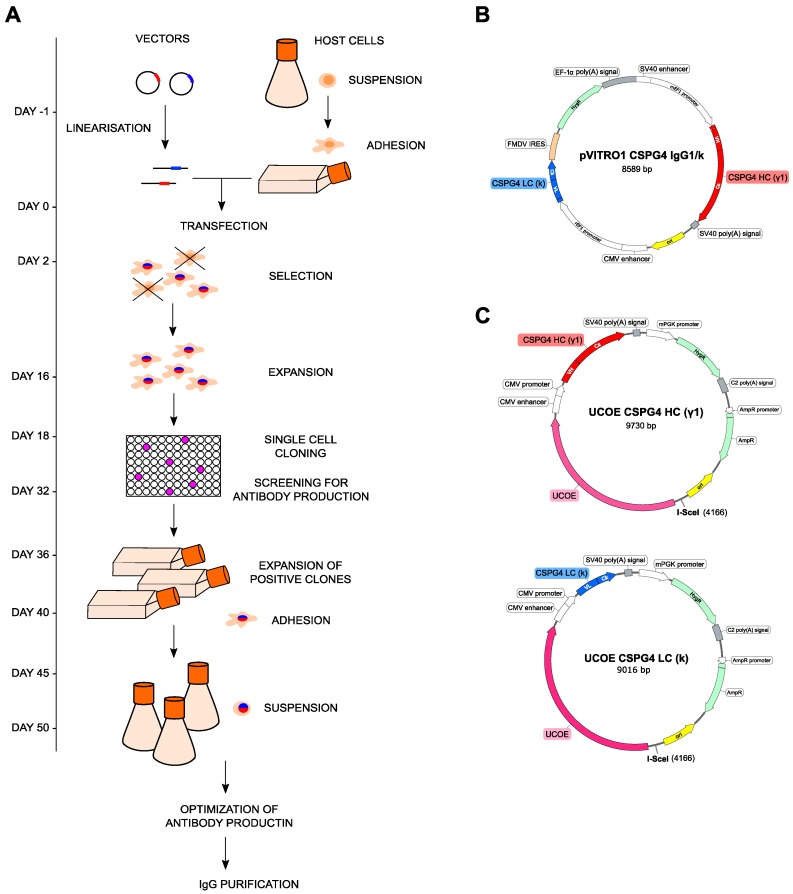
Generation of the anti-CSPG4-IgG1 antibody. (**A**) Schematic diagram summarizing the development of stable cell lines expressing anti-CPSG4 IgG. Vectors were linearized before transfection to allow correct integration into the host genome, and transgene-expressing cells were selected; (**B**) pVITRO1-CSPG4-IgG1/κ vector map; (**C**) UCOE-CSPG4-HC(γ1) and UCOE-CSPG4-LC (κ) vector maps.

We aimed to generate and study an antibody linked to a DNA-interactive mono-alkylating payload, PDD, recognizing a tumor-associated antigen for the treatment of malignant melanoma. First, we engineered a mouse/human chimeric IgG1 antibody recognizing the melanoma-associated antigen CSPG4 (anti-CSPG4 IgG1), cloning the variable regions of a murine IgG2a clone (225.28s) into a human IgG1 backbone [32]. We then developed a stable expression system for rapid production of the recombinant anti-CSPG4 IgG antibody, equivalent to one previously described for the generation of an IgE class antibody [33] (Figure 1A). We isolated the coding sequences of anti-CSPG4 IgG heavy and light chains from a previously described (pVITRO1-CSPG4 IgG1/k) vector (Figure 1B) [32]. We cloned these into 2 UCOE vectors, UCOE-CSPG4-HC[γ1] and UCOE-CSPG4-LC[κ] (Figure 1C) using polymerase incomplete primer extension (PIPE) cloning, and used these vectors to stably transfect Expi293F human embryonic kidney cells. Before transfection, Expi293F cells were adapted from suspension to adherent growth conditions, and adherent cells were then transfected and seeded in selection media to promote host genome integration of exogenous DNA. Resistant cells were cloned by limiting dilution, and clones with high antibody expression in culture supernatants were amplified and re-adapted to grow in high-density suspension cultures in order to harvest antibodies. The choice of the human embryonic kidney cell line variant Expi293F as host was based on human-like antibody glycosylation profiles, ability of the cells to adapt to growth in suspension and adherent formats, and high-density and serum-free conditions. These characteristics are desirable for expediting production, scaling up, and adaptability to good manufacturing practice conditions to facilitate future translation to the clinic. 

**Figure 2:**
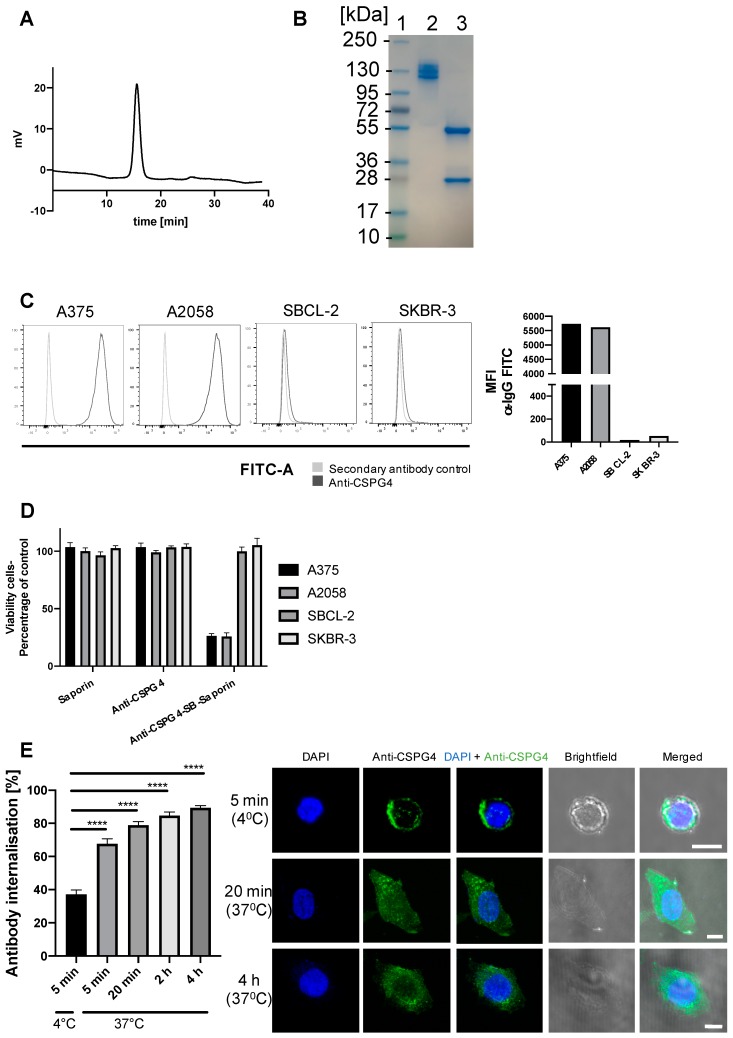
Anti-CSPG4-IgG1 antibody characterization and investigation of target-specific internalization. (**A**) HPLC trace of purified anti-CSPG4-IgG1 antibody; (**B**) SDS-PAGE followed by InstantBlue^®^ protein staining 1 (Expedeon, UK): Molecular weight standard 2: anti-CSPG4 antibody, 3: anti-CSPG4 antibody reduced; (**C**) Flow cytometric analyses (histograms and mean fluorescence intensity (MFI) values) of binding of anti-CSPG4 antibody to CSPG4-high and CSPG4-low cancer cell lines; (**D**) Investigation of cell viability of melanoma cell lines with high (A375, A2058) and low (SBCL-2, SKBR-3) CSPG4-expression. Tumor cells were treated with anti-CSPG4-Saporin, the naked antibody anti-CSPG4 and Saporin alone; (**E**) Investigation of anti-CSPG4 antibody internalization in the melanoma cell line A375 at different time points by confocal microscopy, error bars represent SEM, significance determined by one-way ANOVA with Dunnett’s multiple comparisons test; **** *p* < 0.0001; Scale bar 10 μm, 40× magnification.

Intact full length anti-CSPG4-IgG1 antibody was successfully expressed, with high purity (>95%) as shown by HPLC analyses (Figure 2A). The antibody also had the anticipated molecular weight characteristics as shown by SDS-PAGE gel electrophoresis (Figure 2B). Under non-reducing conditions multiple bands ~150 kDa can be observed but under reducing conditions two bands are observed representing the heavy (~ 55 kDa) and the light (~25 kDa) chains of the antibody.

To engender selective cytotoxicity for target cells, ADCs need to: a) recognize a tumor antigen expressed at higher levels by cancer cells compared with healthy cells and b) to be internalized by the target cells upon recognizing the antigen in order to expose the cell to the toxic payload. CSPG4-expression on target cells was confirmed by flow cytometry (Figure 2C). To evaluate targeting cancer cells with our ADC, we selected CSPG4 high-expressing melanoma cells (A375, A2058) and CSPG4 low-expressing melanoma (SBCL-2) and breast cancer (SKBR-3) cell lines. To confirm that the antibody was internalized by cancer cells, a reporter assay was employed for which the anti-CSPG4 IgG1 was linked to streptavidin and then conjugated to biotinylated Saporin (anti-CSPG4-SB-Saporin). Saporin is a 30 kDa ribosome-inhibitor unable to cross a cell membrane unaided, however Saporin is only toxic once taken up by cells, a process known to happen when it is conjugated to an internalizing antibody, as previously described [34,35]. Treatment with anti-CSPG4-SB-Saporin for 4 days decreased tumor cell viability in CSPG4-high A375 and A2058 melanoma cell lines, while it had low toxic effects on the CSPG4-low SBCL-2 melanoma and SKBR-3 breast cancer cells. 

As expected, none of the cell lines studied showed any loss in cell viability when treated with naked antibody or with Saporin alone (Figure 2D). In concordance, we confirmed antibody internalization by A375 melanoma cells in a time-dependent manner by confocal microscopy analysis of fluorescently labelled anti-CSPG4 antibody (Figure 2E). Together the reporter and imaging findings suggest that anti-CSPG4-IgG1 internalization occurred in CSPG4- expressing melanoma cells. These data confirmed the generation of intact anti-CSPG4-IgG1 able to be internalized in CSPG4-high expressing melanoma cells, but less so in CSPG4-low expressing melanoma or breast cancer cell lines.

### 2.2. Evaluation of Payload Toxicity across Different Cancer Cell Types

**Figure 3:**
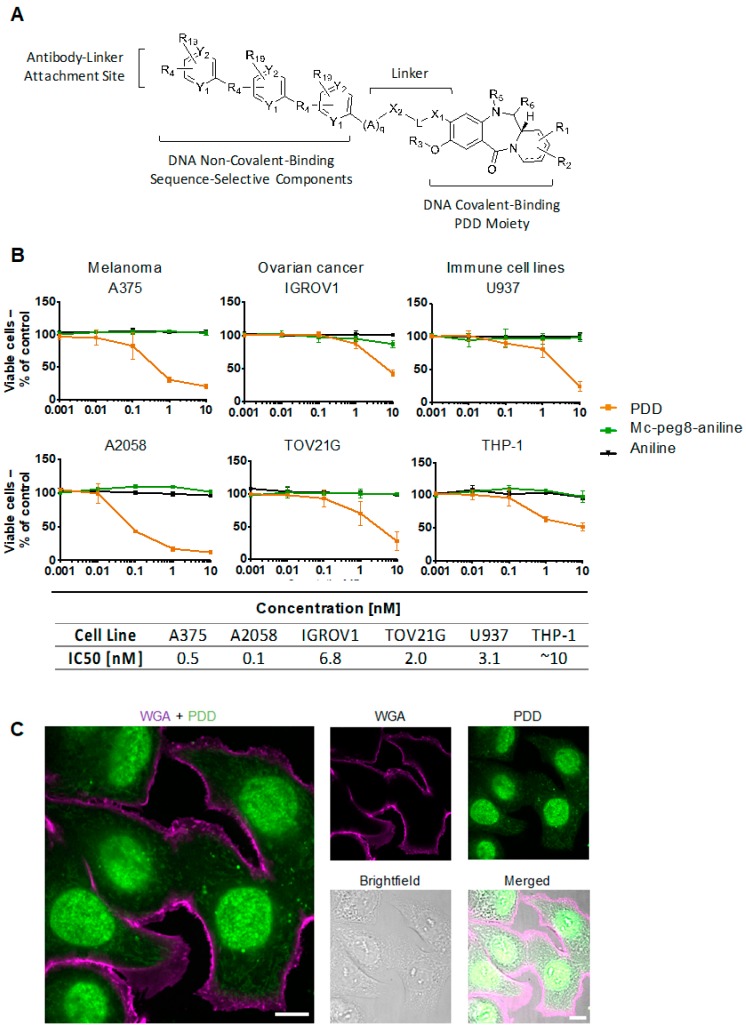
Structure, cytotoxicity profile and localization of the novel payload PDD. (**A**) Schematic of the PDD-based payload consisting of an antibody-linker attachment site, DNA non-covalent-binding sequence-selective components, linker and DNA covalent-binding PDD moiety; (**B**) Investigation of the cytotoxicity of the PDD in melanoma (A375, A2058), ovarian (IGROV1, TOV21G) and immune (U937, THP-1) cell lines. Cell viability was measured upon treatment with the PDD, a dummy payload (aniline) and the linked dummy payload mc-peg8-aniline; IC_50_ values are shown for each cell line below; (**C**) Investigation of PDD intracellular localization in SKBR-3 cells after 3 hours of incubation by confocal microscopy (scale bar: 10 μm), 100× magnification.

We next investigated the suitability of the PDD (Figure 3A) as a potent payload for this antibody. This molecule is designed to covalently bind to the C2-amino groups of guanine bases in the minor groove of DNA to form mono-adducts. Cell viability assays were performed in different cell types, specifically melanoma (A375, A2058), ovarian (IGROV1, TOV21G) and immune (U937, THP-1) cell lines with the PDD-based agent, a “dummy” payload (aniline) and mc-peg8-aniline (linker-dummy payload). The aim was to assess toxicity of the payload and of controls across different cancer cell and immune cell types. Results showed cytotoxicity for the PDD-based agent only, with IC_50_ values in the low nanomolar to picomolar range across multiple cell target types. As expected, there were no effects on cell viability for aniline or mc-peg8-aniline (Figure 3B). Furthermore, confocal microscopy confirmed the intracellular localization of the PDD in the nucleus of cancer cells after 3 hours incubation (Figure 3C). The results therefore show that the PDD alone affects cell viability in various cancer and monocytic-derived cell lines at different levels (Figure 3B) and may suggest that the efficacy of a PDD-bearing ADC may not only depend on the antibody target expression but also on the potency of the PDD itself. Our findings may also support the use of the PDD as a payload to target melanoma cells due to its picomolar IC_50_ profile in both melanoma cell lines investigated, compared to nanomolar IC_50_ values measured for all other cell lines (Figure 3B). We therefore selected melanoma as a target tumor for an anti-CSPG4 ADC bearing a PDD payload.

### 2.3. Generation of Anti-CSPG4(PDD) ADC by Stochastic Conjugation

**Figure 4:**
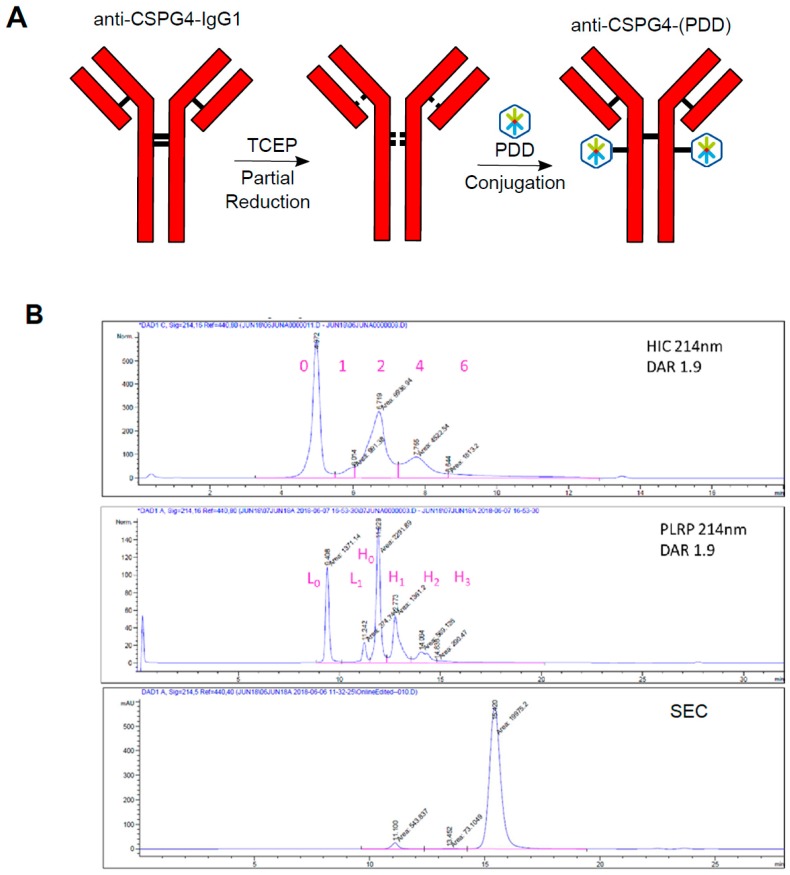
Conjugation of anti-CSPG4-IgG1 antibody to PDD payload via stochastic conjugation generates anti-CSPG4-(PDD) ADC with a drug antibody ratio (DAR) of 1.9. (**A**) Schematic diagram of stochastic ADC conjugation by antibody reduction with TCEP, followed by partial oxidation with DHAA and then conjugation to PDD via a Val-Ala-maleimide linker; (**B**) Analytical data from the stochastic conjugation of the anti-CSPG4-IgG1 antibody to the PDD. The HIC (top panel) and PLRP (middle panel) analyses confirm an average drug-antibody ratio (DAR) of 1.9, and the Size Exclusion Chromatography (SEC) trace (bottom panel) indicates negligible aggregation of the ADC and no detection of free linker-payload.

The anti-CSPG4 IgG1 was conjugated stochastically to PDD through a maleimide-based Val-Ala linker to create the novel anti-CSPG4-(PDD) ADC (Figure 4A). Analysis of the ADC using hydrophobicity interactions chromatography (HIC) and polymeric reversed phase chromatography (PLRP) confirmed that conjugation had occurred with an average drug-antibody ratio (DAR) of 1.9. Furthermore, size exclusion chromatography (SEC) indicated negligible ADC aggregation (Figure 4B). A control non-binding isotype (PDD)-conjugated ADC (isotype-(PDD)) was also prepared (DAR = 2.1) based on a hen egg lysosome IgG1 antibody (Figure S1). SDS-PAGE also confirmed formation of the anti-CSPG4-(PDD) and isotype-(PDD) ADCs with negligible amounts of free light chain. The intact product showed multiple bands around 150 kDa (Figure S2). 

### 2.4. Anti-CSPG4-(PDD) ADC Restricts Viability and Promotes Apoptosis of CSPG4-Expressing Melanoma Cells

**Figure 5:**
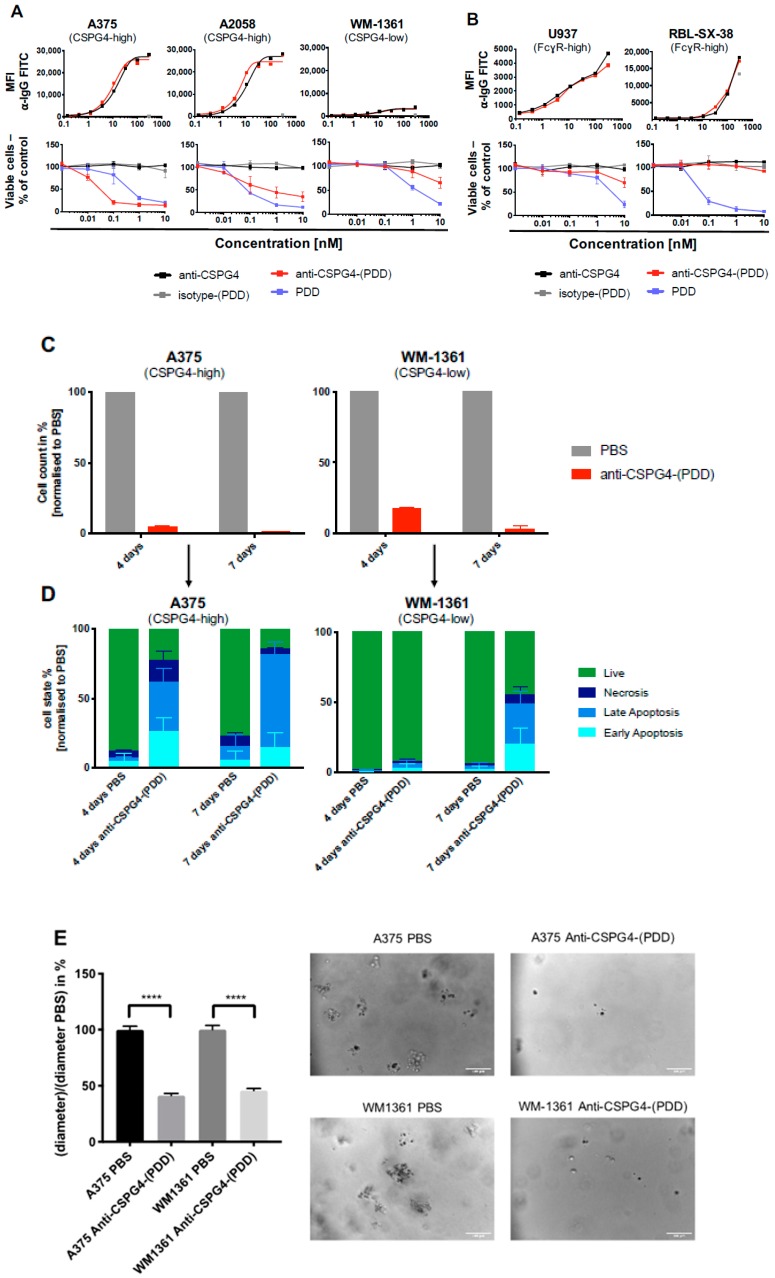
Anti-CSPG4-(PDD) promotes target selective melanoma cell killing and tumor cell apoptosis with no toxicity to immune cell lines. (**A**) Binding of anti-CSPG4 and anti-CSPG4-(PDD) to CSPG4-high and CSPG4-low melanoma cell lines (top panel). Investigation of cell viability of melanoma cell lines with high (A375, A2058) and low (WM1361) CSPG4-expression. Tumor cells were treated with the naked antibody anti-CSPG4, isotype-(PDD) control ADC, anti-CSPG4-(PDD) ADC or PDD alone; (**B**) Binding of anti-CSPG4 and anti-CSPG4-(PDD) monocytic cell line U937 and basophil-derived cell line RBL-SX-38 with high Fcγ-receptor expression (top panel). Investigation of cell viability of U937 and RBL-SX-38 cells treated with naked antibody anti-CSPG4, isotype-(PDD) control ADC, anti-CSPG4-(PDD) ADC or PDD alone. *N* = 1 for all binding assays and *N* = 3 independent experiments for all MTS studies, each condition performed in triplicate, error bars represent Standard Deviation (SD); (**C**) Cell count of CSPG4-high A375 and CSPG4-low WM-1361 cells post treatment with anti-CSPG4-(PDD) ADC or PBS control. Cell counts were evaluated using Flow Cytometry counting beads; (**D**) Analysis of cell state of CSPG4-high A375 and CSPG4-low WM-1361 cells 4- and 7-days post treatment with anti-CSPG4-(PDD) ADC or PBS control; (**E**) Analysis (left) and images (right) of colony formation of CSPG4-high A375 and CSPG4-low WM-1361 cancer cells following 7-day treatment with anti-CSPG4-(PDD) ADC or PBS control. *N* = 3 independent experiments, error bars represent Standard Deviation (SD). Scale bar: 100 μm; **** *p* < 0.0001.

We next evaluated whether the ADC could selectively kill antigen-expressing tumor cells while showing little or no toxic effects towards low target-expressing cells. Firstly, we demonstrated similar binding kinetics of the unconjugated anti-CSPG4 antibody compared with the anti-CSPG4-(PDD) ADC in three melanoma cell lines with high and low expression of CSPG4. As expected, we detected no binding of the isotype-(PDD) control ADC in any cell line (Figure 5A, top panel). To investigate the potential efficacy of the anti-CSPG4-(PDD) ADC, its effect was evaluated on melanoma cells expressing high and low levels of the CSPG4 target antigen using the naked antibody anti-CSPG4, PDD and the non-binding isotype-(PDD) ADC as controls (Figure 5A, bottom panel). 

In high CSPG4-expressing A375 and A2058 human melanoma cells, anti-CSPG4-(PDD) as well as PDD alone significantly reduced cell viability at low nanomolar levels after 4 days. In CSPG4-low WM1361 human melanoma cells, anti-CSPG4-(PDD) caused a slight decrease in cell viability at the highest concentration only (10 nM). In contrast, the unconjugated PDD payload exerted a concentration-dependent decrease in cell viability, independent of antigen expression levels. As expected, neither the anti-CSPG4 antibody nor isotype-(PDD) ADC had any effects on cell viability in any of the three melanoma cell lines. These findings indicated that the ADC could deliver the PDD to CSPG4-expressing cancer cells in a target-specific manner. The mechanism of ADC payloads dissociating from the antibody has been described previously, reporting that upon ADC internalization, cleavable dipeptide linkers such as the herein used Val-Ala-maleimide linker get cleaved by peptidases in the lysosome, thereby releasing the payload [36].

To evaluate target selectivity, next the viability of non-CSPG4 expressing cells was investigated towards anti-CSPG4-(PDD) ADC, isotype-(PDD) ADC, naked anti-CSPG4 antibody and the PDD payload alone in FcγR-high monocytic U937 and the basophilic leukemia RBL-SX-38 cells (Figure 5B). Comparable levels of Fc-mediated binding of the monoclonal antibody anti-CSPG4 IgG1 and the ADC anti-CSPG4-(PDD) were observed in both cell lines, confirming that stochastic conjugation with the payload did not impair the antibody’s ability to engage with Fc-receptor-expressing cells (Figure 5B, top panel). As anticipated, the PDD alone reduced cell viability in both cell lines in a concentration-dependent manner. However, once conjugated to anti-CSPG4 IgG1, the anti-CSPG4-(PDD) ADC had no impact on the viability of U937 or RBL-SX-38 cells (Figure 5B, bottom panel). These findings confirmed that conjugation of the antibody to the PDD did not impair Fc region-mediated binding of the ADC to immune cells, and this Fc-mediated interaction did not induce cytotoxicity towards these immune cells. 

Next, to gain an insight into the potential mechanism by which anti-CSPG4-(PDD) exerted cytotoxicity towards melanoma cells, CSPG4-high A375 and CSPG4-low WM-1361 cells were quantified after four and seven days of treatment with PBS and anti-CSPG4-(PDD) (Figure 5C). These analyses revealed fewer cells in both melanoma cell lines compared to PBS controls, and more cells in the CSPG4-low WM-1361 cells (~17% compared to PBS) compared to the CSPG4-high A375 cells (~5% compared to PBS) 4 days after ADC treatment. In both cell lines after 7 days of treatment with ADC, we measured <5% of cells compared to PBS controls, suggesting that anti-CSPG4-(PDD) inhibited cancer cell proliferation during this extended time. The remaining cells were then subjected to Annexin V and DAPI staining in order to determine the proportions of the remaining cells in early apoptosis (Annexin V+, DAPI-), late apoptosis (Annexin V+, DAPI+), necrosis (Annexin V-, DAPI+) or live (Annexin V-, DAPI-) (Figure 5D).

After four days of treatment with the ADC, 77% of remaining CSPG4-high A375 cells were shown to be in early or late apoptosis or necrosis, while 92% of CSPG4-low WM-1361 cells were still detected as live. After seven days of ADC treatment, from the 14% of A375 cells detected, 67% were found to be in late apoptosis. At the same time point, ~44% of CSPG4-low WM-1361 cells were detected as live cells with ~20% in early apoptosis and ~28% in late apoptosis. These results suggest shutdown of cell proliferation upon anti-CSPG4-(PDD) treatment (Figure 5C), followed by apoptotic cell death, timed to be consistent with expression levels of the target antigen CSPG4. This may be because lower target antigen expression in WM-1361 cells may have led to less ADC internalization compared to CSPG4-high A375 cells, causing WM-1361 cells to enter apoptosis later than A375 cells. To evaluate whether anti-CSPG4-(PDD) can restrict colony formation of melanoma cells, cell colonies were measured following treatment with PBS and anti-CSPG4-(PDD) for seven days in the CSPG4-high and CSPG4-low A375 and WM-1361 cells, respectively (Figure 5E). These revealed significantly reduced colony formation in both melanoma cell lines compared to PBS controls; CSPG4-high A375 cells ~41% colony size compared to PBS, CSPG4-low WM-1361 cells ~46% colony size compared to PBS, both 7 days after ADC treatment. Together, these findings indicated that anti-CSPG4-(PDD) can exert Fab-mediated cytotoxicity towards melanoma cells in an antigen-specific manner, with lower Fc-mediated cytotoxicity to immune cells. Anti-tumor effects included shutdown of melanoma cell proliferation and colony growth and onset of apoptosis in melanoma cells.

### 2.5. Anti-CSPG4-(PDD) Displays Tumor-Growth Restricting Potency in Vivo

**Figure 6:**
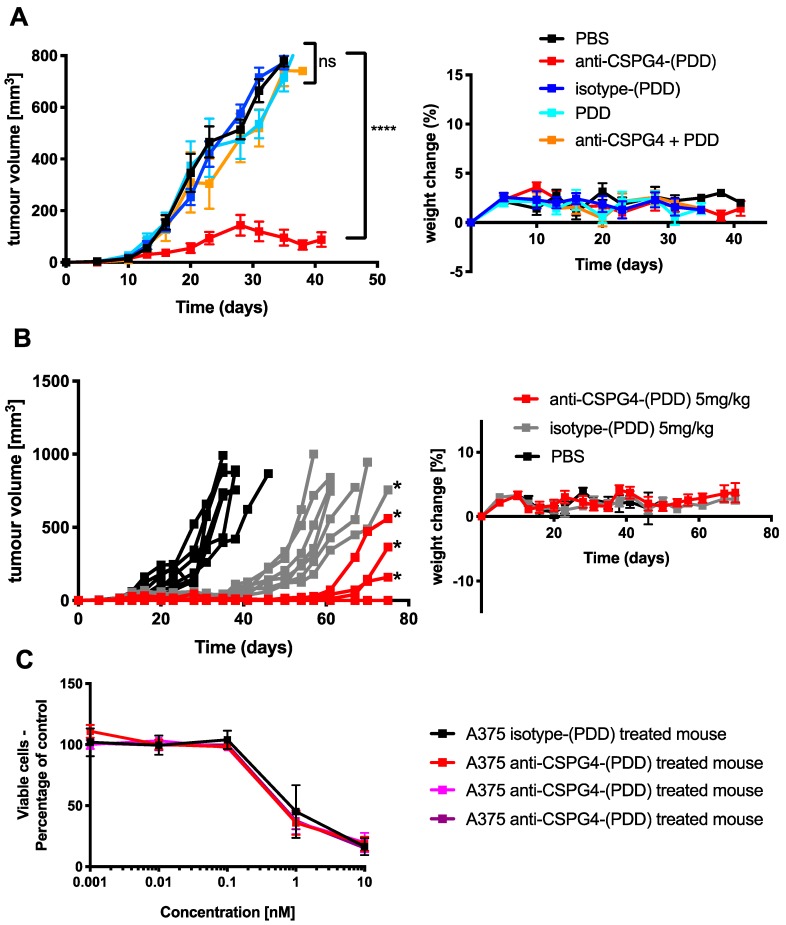
Anti-CSPG4-(PDD) restricts tumor growth in vivo in a melanoma xenograft with low toxicity profile. (**A**) Growth curves of A375 human tumor xenografts in female athymic nude mice (mean ± SEM, left panel) and mouse weights (mean ± SD (right panel, *N* = 7 mice per group), treated with anti-CSPG4-(PDD) ADC, isotype-(PDD) control ADC, PDD alone, a combination of the anti-CSPG4 antibody and non-conjugated PDD or PBS vehicle control with two doses of 2 mg/kg at days 10 and 24 post tumor inoculation. (**** *p* < 0.0001, by Dunnett’s multiple comparisons test). (**B**) Growth curves of A375 human tumor xenograft in female athymic nude mice (single lines [left panel]; mouse weight mean ± SD (right panel); *N* = 7 mice per group, treated with anti-CSPG4-(PDD) ADC, isotype-(PDD) control ADC, or PBS vehicle control with one dose of 5mg/kg at day 10 post tumor inoculation, * indicates harvested tumors used for ex vivo study in (**C**) below, (**** *p* < 0.0001, by Dunnett’s multiple comparisons test). (**C**) Ex vivo cell viability (MTS) of A375 cells from anti-CSPG4-(PDD)- and isotype-(PDD)-treated mice (harvested tumors marked by *). Tumor cells were treated with anti-CSPG4-(PDD) (*N* = 3 independent experiments, each condition performed in triplicate, error bars represent standard deviation [SD]).

To evaluate the anti-tumor activity of the anti-CSPG4-(PDD) ADC, a human melanoma xenograft grown subcutaneously in athymic nude mice was established (Figure 6A). Two doses of 2 mg/kg per mouse were administered (i.v.) on days 10 and 24 (following tumor inoculation) in A375 melanoma human tumor xenograft-bearing mice.

A significant reduction of tumor growth was observed at this dose level (tumor size at the end of study +/− SEM per group in mm^3^: PBS 771 ± 10, anti-CSPG4-(PDD) 88 ± 28, isotype-(PDD) 770 ± 11, PDD alone 791 ± 18, anti-CSPG4 + PDD 787 ± 12) (*p* < 0.0001, Dunnett’s multiple comparisons test) (Figure 6A, left panel), and below the Maximum Tolerated Dose (MTD) (Figure S3). As anticipated, treatment of the xenograft mice at the same dose level with the equivalent non-binding isotype-(PDD) control ADC, the unconjugated anti-CSPG4-IgG1 antibody plus free payload, PDD alone or PBS vehicle control did not impair tumor growth. Crucially, no overt toxicity was observed in any of the mice (Figure 6A, right panel). These findings indicate the ability of anti-CSPG4-(PDD) to significantly restrict tumor growth in vivo to antigen-expressing tumor cells compared with control treatments which included non-specific isotype control ADC, and free antibody plus free payload combined treatments.

To investigate whether anti-CSPG4-(PDD) had the potential to abolish, rather than restrict tumor growth with one dose, a further in vivo study was performed in which athymic nude mice were challenged with A375 subcutaneous human melanoma xenografts and then treated with 5 mg/kg anti-CSPG4-(PDD), PBS or isotype-(PDD) ADC control. Treatments were administered (i.v.) on day 10 following tumor inoculation. Unlike PBS controls, complete tumor growth arrest was observed in four out of seven mice treated with a single dose of anti-CSPG4-(PDD). In the three remaining mice of this group, tumor growth resumed after 50 days following treatment (tumor size at end of study +/- SEM per group in mm^3^: PBS 836 ± 31, anti-CSPG4-(PDD) 155 ± 85, isotype-(PDD) 834 ± 38) (Figure 6B, left panel). As previously reported [37], we also observed at this higher dose, the isotype-(PDD) partly decelerated tumor growth compared to vehicle control (PBS) treated mice. Crucially, no overt toxicity was observed in any of the mice (Figure 6B, right panel). 

Finally, to investigate whether developed tumors in mice could still respond to anti-CSPG4-(PDD), developed tumors from the three mice were harvested alongside a tumor from an isotype-(PDD)-treated mouse (Figure 6B). Extracted melanoma cells were treated with Anti-CSPG4-(PDD) ex vivo. A375 tumor cells harvested from the isotype-(PDD)-treated mouse and from the anti-CSPG4-(PDD)-treated mice were equally susceptible to ex vivo treatment with anti-CSPG4-(PDD). This suggested that cancer cells from these tumors showed no signs of resistance to the toxic effects of anti-CSPG4-(PDD) (Figure 6C). 

Overall, these results provided evidence in support of combining the payload PDD with the anti-CSPG4-IgG1 antibody to generate an ADC with tumor growth-restricting properties in vitro and in vivo in the absence of overt systemic toxicities. 

## 3. Discussion

ADCs have emerged as promising new therapeutic agents capable of delivering highly-cytotoxic payloads directly to the tumor site. Since there are no clinically-available antibodies directly targeting melanoma cells, and half of patients with melanoma do not respond or develop resistance to approved therapies, we sought to develop an ADC based on a novel payload class to which melanoma cells may be susceptible, using an antibody that recognizes a cell surface antigen expressed by a large proportion of melanomas. 

Appropriate target selection is crucial for the development of novel ADCs. Therefore, we produced an antibody recognizing the tumor-associated antigen CSPG4, as this cell surface neural crest antigen is over-expressed in a large proportion (~70–80%) of melanotic and other solid tumors, and it is found on cells likely to confer an aggressive phenotype [20,22,23]. We confirmed target antigen recognition by the antibody and its ability to be internalized by antigen-expressing tumor cells using a reporter assay. Previous studies have shown that CSPG4-expressing tumors could be targeted with CSPG4-specific agents [38,39,40,41]. This included the monoclonal antibody Ep1 to deliver cisplatin encapsulated in protein cages [42]. This CSPG4-targeted treatment showed target-specific cell killing and a delay in tumor growth in vivo [42]. CSPG4-targeting antibodies have also been used to deliver immunotoxins to CSPG4-expressing melanoma cells, which were then released from endo-lysosomal compartments into the cytosol by photochemical internalization [43]. Delivering immunotoxins to solid tumors might be limited by restricted transport of large molecules across the vascular endothelium, and poor light penetration in pigmented melanoma lesions [43]. However, these studies provided promising findings in support of targeting CSPG4 with antibodies and conjugated agents.

Here, our approach is based on coupling a novel PDD-based DNA minor groove-binding agent to an in-house engineered anti-CSPG4 antibody. This resulted in target antigen-specific melanoma cell killing at low antibody and drug doses in the low nanomolar to picomolar range as well as an efficient restriction of tumor growth in vivo. The PDD payload is structurally-related to the PBDs. However, unlike the PBD dimers it does not cross-link DNA but instead works by forming mono-alkylated guanine adducts in the DNA minor groove. Currently, 29 ADCs have been developed based on a PBD dimer payload, 20 of which have entered clinical development, with nine in active clinical trials and 11 discontinued. PBD-based DNA cross-linking agents have been linked to concerns regarding on- and off-target toxicities due to non-specific uptake of the payloads into sensitive normal tissues, resulting in acute and delayed toxicity in both pre-clinical and clinical settings [44]. Toxicity issues led to the development of a similar class of payloads that still bind in the DNA minor groove but form mono-alkylated adducts instead which are less systemically toxic, potentially due to their ability to be more easily repaired by healthy cells. Examples of mono-alkylating DNA-interactive payloads include the indolinobenzodiazepines (IGNs) and the PDDs [44]. Due to the mono-alkylating mechanism of action of the PDDs, there is evidence to suggest that they may enhance the therapeutic window of ADCs by reducing off-target cytotoxic effects. Consistent with this notion, previous work comparing the site-specific THIOMAB conjugation of a PDD and the PBD dimer Talirine to trastuzumab resulted in an MTD of 4mg/kg for the THIOMAB-PBD dimer and an MTD of > 8mg/kg for the THIOMAB-(PDD) ADC [45]. In this treatment setting, the anti-CSPG4-(PDD) administered in two 2 mg/kg doses significantly restricted tumor growth compared with isotype-(PDD) ADC, PDD payload alone, anti-CSPG4 plus unconjugated PDD or buffer control-treated groups. These findings suggest that an effective dose would be significantly below the MTD, indicating a significant Therapeutic Index (TI) for this ADC. Future experiments will be required to evaluate the efficacy and safety of anti-CSPG4-(PDD) in low-CSPG4 expressing tumor models in vivo. 

Furthermore, a single injection of 5 mg/kg of anti-CSPG4-(PDD) led to complete tumor regression of A375 xenografts in 4/7 mice for 70 days following treatment in the absence of overt toxicities. At this higher dose, the isotype-(PDD) can exert some tumor growth restricting effects, a phenomenon previously observed by others when administering isotype-ADCs at higher doses [37]. Interestingly, melanoma cells harvested from the three mice that responded for 50 days after ADC treatment remained sensitive to anti-CSPG4-(PDD) treatment ex vivo compared with A375 tumor cells harvested from the isotype-(PDD) treatment mouse. This suggested that recurrent tumors had not developed resistance to the ADC, meaning that they may be: a) susceptible to drug and b) targeted by the ADC in an antigen-specific manner. These findings may be significant, since resistance can develop against the antibody or the payload components of the ADC by antigen down-regulation or mutation, or by upregulation of drug efflux transporters, respectively. Resistance has been observed with multiple ADCs in clinical use [2] which has restricted efficacy. For instance, gemtuzumab ozogamicin has been associated with multidrug resistance (MDR) mechanisms [46]. Resistance mechanisms towards brentuximab vedotin have been investigated, since more than a third of treated patients that initially achieve a complete response, relapsed and died within five years of treatment [2]. CD30 down-regulation, MMAE resistance, and upregulation of the MDR1 drug exporter protein have all been shown to be associated with resistance to brentuximab vedotin [47]. However, our data are consistent with previous reports of low nM to pM cytotoxicity for PDDs against a panel of tumor cell types, including MDR-resistant tumors [45]. From the antigen-antibody targeting perspective, CSPG4 may promote chemoresistance by activation of integrin-dependent PI3K/Akt signaling in cell lines and patient samples [48]. However, CSPG4 has been suggested to be less prone to antigen loss during CAR-T-cell therapy [21], consistent with our findings that residual tumor cells remain susceptible to anti-CSPG4-PDD treatment. This may signify that our anti-CSPG4-(PDD) ADC might be less prone to resistance in relation to antigen down-regulation and payload efflux. 

When melanoma, ovarian and immune cell lines were treated with nanomolar and picomolar concentrations of PDD, along with the structurally-similar dummy payload aniline, and aniline conjugated to a maleimide-peg linker (mc-peg8-aniline), only the PDD gave a concentration-dependent decrease in cell viability in all cell lines with especially high toxicity effects on melanoma cells. While we expected the PDD payload to be universally toxic to different cell types, conjugation to an antibody may be used to drive toxic effects specifically against high antigen-expressing cancer cells, potentially sparing normal cells. The novel anti-CSPG4-(PDD) ADC was therefore produced using a Val-Ala-maleimide linker with DAR 1.9 (confirmed by HIC and PLRP) and negligible amounts of aggregates (shown by Size Exclusion Chromatography following purification). The presence of multiple bands under non-reducing conditions detected by SDS-PAGE of purified anti-CSPG4 antibody (Figure 2b) and anti-CSPG4-(PDD) (Figure S2) might represent different glycoforms of the antibody, whilst HIC, PLRP and SEC (Figure 4b) showed production of a clean ADC product. We showed comparable CSPG4 recognition profiles between anti-CSPG4-(PDD) ADC and the unconjugated anti-CSPG4 antibody in all three target cell lines studied, suggesting that payload conjugation had no impact on Fab-antigen recognition (Figure 5A). Tumor cell viability was reduced in melanoma cell lines with high CSPG4 expression (A375, A2058) when treated with anti-CSPG4-(PDD), whereas a lower reduction in cell viability could be observed in the CSPG4-low WM-1361 cell line. These findings suggested that appropriate dosing of this ADC is important. None of the cell lines showed reduced cell viability upon treatment with naked antibody or isotype-(PDD). This was expected since: a) concentrations of the naked antibody tested were too low to observe any direct anti-tumor effects, b) the lack of tumor antigen recognition of the isotype control antibody prevented targeting of the payload to cancer cells and triggering any cell death, and c) the linker stability of the isotype-(PDD) prevented the release of extracellular PDD to allow entry to the cell to trigger cell death. These findings suggest that once comparatively low amounts of drug are conjugated to an internalizing antibody any toxic effects are then directed to tumor antigen-expressing cells. These data point to significantly altered pharmacokinetic properties of the toxic payload towards high target antigen-expressing but less towards low target antigen-expressing tumor cells when the payload is attached to a tumor antigen-specific antibody. Overall, the anti-CSPG4-(PDD) conjugate is expected to internalize inside cancer cells that express the target antigen CSPG4 (Figure 2). Once inside the cell, the cytotoxic payload (i.e., the PDD) may be cleaved from the antibody by protease enzymes in the lysosome and the drug translocates to the nucleus (Figure 3), where it mono-alkylates the DNA. This could cause a shutdown of melanoma cell proliferation within 3-4 days, which may be more pronounced in cells with high CSPG4 expression and also in low CSPG4-expressing cells through apoptosis and reduction in colony formation after 7 days (Figure 5). It is possible that the effects of the ADC on cancer cells may also depend on target antigen expression, the potency of the payload as well as the length of exposure to the drug. 

An ADC introduced into a patient’s circulation will inevitably encounter not only tumor cells, but also a range of non-target antigen-expressing, and also Fc Receptor-expressing immune cells. We therefore investigated the possibility of an ADC binding through its Fc domains and triggering toxic effects to immune cells. Fcγ Receptor binding and internalization studies of anti-CSPG4-(PDD) showed no significant toxicity to immune cells, in a similar manner to naked antibody- or isotype-(PDD) control-treated cells. This suggested that no Fc-mediated internalization or off-target cytotoxicity was occurring with the anti-CSPG4-(PDD) ADC. This is especially important since hepatic toxicities have been reported for several ADCs, often driven via carbohydrates, specifically a-galactosylated glycans, that interact with immune cells [49]. This may be significant in the field of ADCs since it is possible that the antibody Fc regions of ADCs could engender Fc-mediated toxic effects, although this has been insufficiently generally evaluated [50]. On the other hand, when we investigated the tumor cell killing effects of anti-CSPG4-(PDD) in CSPG4-high expressing A375 and CSPG4-low expressing WM-1361 melanoma cells, we observed a time- and target-antigen dependent loss in proliferation in anti-CSPG4-(PDD)-exposed cells compared to PBS controls. This was accompanied by apoptotic cell death rates higher and faster in high CSPG4-expressing cells compared with low target-expressing cells. Our data suggest that anti-CSPG4 and PDD may be a suitable antibody and payload combination for the development of an anti-CSPG4-(PDD) ADC with potent target antigen-specific cytotoxic functions via the ability to trigger apoptotic cell death in CSPG4-expressing melanoma cells, whilst the ADC lacked off-target or Fc-mediated cytotoxic effects. 

## 4. Materials and Methods 

### 4.1. Cell Lines

Expi293F cells were purchased from Thermo Fisher Scientific (Loughborough, UK), IGROV1 cells were kindly gifted by Professor Silvana Canevari and Dr Mariangela Figini (Instituto Nazionale Tumori, Milan, Italy), the human melanoma WM-1361 cells and monocytic THP-1 cells were kindly gifted by Professor Victoria Sans-Moreno (Queen Mary University, London, UK) and all other cell lines were purchased from ATCC. Expi293F cells were cultured in Expi293 Expression Medium (A1435101, Thermo Fisher Scientific) in suspension and shaking conditions (125 rpm) at 37 °C in an 8% CO_2_ atmosphere, according to the manufacturer’s instructions. A375, A2058 and WM-1361 melanoma cells as well as SKBR-3 cells were cultured in adhesion in Dulbecco modified Eagle medium (DMEM) high glucose supplemented with GlutaMAX (10566016, Gibco, Loughborough, UK) and 10% FBS. IGROV1, TOV-21G ovarian and U937, THP-1 immune cells were cultured in adhesion in Roswell Park Memorial Institute (PRMI) high glucose supplemented with GlutaMAX (61870010, Gibco, Loughborough, UK) and 10% FBS. Cells once tested negative for mycoplasma were used up to 20 passages. 

### 4.2. CSPG4 IgG Expression 

The pVITRO1-CSPG4-IgG/k vector (Figure 1B) was previously developed in our group [32]. The dual UCOE-vector system (UCOE-CSPG4-HC[ε] and UCOE-CSPG4-LC[κ]; Figure 1C) was developed by using PIPE cloning, as described previously for the IgE version of this antibody [33]. A stable anti-CSPG4 IgG-expressing Expi293F cell line has been developed as previously described for the anti-CSPG4 IgE [33]. Anti-CSPG4 IgG used in this study was purified into PBS using Protein A columns (Thermo Fisher Scientific, 20356) according the manufacturer’s instructions, followed by analytic size exclusion chromatography as described below.

### 4.3. Size Exclusion Chromatography

Purified antibodies were analyzed by using size exclusion chromatography, as previously described [51]. Briefly, gel filtration was performed on a Gilson HPLC system (Gilson, Dunstable, UK) using a Superdex 200 10/300 GL column (GE Healthcare, Buckinghamshire, UK), which is suitable for purifying proteins between 10 and 300 kDa at a flow rate of 0.75 mL/min in PBS (pH 7.0, 0.2 μm filtered). 

### 4.4. Antibody-Drug Conjugate Production and Characterization

Anti-CSPG4 IgG1 and hen egg lysosome IgG1 have been stochastically conjugated to PDD via a Val-Ala-maleimide linker to produce anti-CSPG4-PDD and isotype-(PDD) ADCs. Briefly, the interchain disulfides of both antibodies, formulated pH 7- 8 PBS 2 mM EDTA, were partially reduced with the mild reductant TCEP for 90–180 minutes. The extent of reduction was controlled to achieve a drug-to-antibody ratio (DAR) 2. The reduced antibody was diluted with PBS 2 mM EDTA to 2 mg/mL PDD-Ala-Val-maleimide was dissolved in 10 mM DMSO. Conjugation of the antibodies was achieved by addition of an excess of the payload to a 1:1 mixture of the reduced antibody and propylene glycol, at a final protein concentration of 1 mg/mL. The antibodies and the PDD-Ala-Val-maleimide were conjugated for 1hr to form the ADCs anti-CSPG4-(PDD) and isotype-(PDD) and then the reactions quenched with an excess of N-acetylmaleimide. The ADCs were further diluted 1:1 with PBS 3% cyclodextrin, then bound to a Protein A resin. The resin-bound ADCs were washed with PBS 3% cyclodextrin to remove excess small-molecule impurities, then released from the resin. The ADC was formulated through G25 desalting into PBS 3% cyclodextrin and 0.2 µm filtered prior to aliquoting and −80 °C storage. ADCs were characterized using hydrophobicity interaction chromatography (HIC), polymeric reverse phase chromatography (PLRP) and size exclusion chromatography (SEC) which provided information on overall purity, drug antibody ratio (DAR) and the degree of aggregate formation.

### 4.5. DAR by Hydrophobic Interaction HPLC (HIC)

HIC was performed on a TOSOH Butyl-NPR 4.6 mm x 3.5 cm, 2.5 µm column (Tosoh Corp., Tokyo, Japan) at 0.8 mL/min with a 12-minute linear gradient between mobile phase A - 1.5 M (NH_4_)_2_SO_4_, 25 mM NaPi, pH = 6.95 ± 0.05 and MOBILE PHASE B - 75% 25 mM NaPi, pH 6.95 ± 0.05, 25% IPA. Samples were loaded neat up to a maximum loading of 10 µl and data collected at 280 and 214 nm; all reported data are 214 nm. 

### 4.6. Residual Toxin Linker by Reverse Phase HPLC (RPLC)

Analysis was performed on a Kinetex Core Shell 2.6 µm column (Phenomenex, Macclesfield, UK, C_8_, 100 Å, 50 × 4.6 mm) run at 2 mL/min/60 °C with a linear gradient between 95% A (0.05% TFA/H_2_O) to 95% B (0.05% TFA/CH_3_CN). NAC-quenched toxin linker standards were run as 100, 50, 25, 10, and 5 pmol samples in methanol. Data were collected and analyzed at 214 nm.

Conjugate samples were deproteinated by cold methanol extraction prior to HPLC analysis: 2 µL of 5 M NaCl were added to 50 µL of sample, followed by 150 µL of ice-cold methanol. Samples were vortexed and incubated at −20 °C for 30 minutes. Precipitated protein was pelleted by centrifugation at 15,000 × g for 30 minutes at 4 °C. Next, 125 µL of the supernatant were diluted with 125 µL Elga 18.2 MΩ water and mixed. A 100 µL sample was injected in duplicate.

### 4.7. Monomer Content and Protein Concentration by Size Exclusion HPLC (SEC)

The aggregate content of each conjugate was assessed by chromatography on a TOSOH TSKgel G3000SWXL 7.8 mm × 30 cm, 5 µm column at 0.5 mL/min in 10% IPA, 0.2M potassium phosphate, 0.25 M potassium chloride, pH 6.95. Samples were loaded neat and data collected at 214, 252 and 280 nm. All reported data are at 214 nm.

For the determination of protein concentration, the antibodies were injected at various volumes (0.5, 1, 2, 3, 4, and 5 µL) and the area under the curve at 214 nm recorded. A linear regression model (area = concentration × slope + intercept) was fitted to the data and used to calculate the concentration of the antibody-drug conjugates.

### 4.8. SDS-PAGE Gel Electrophoresis

4–15% Mini-Protean TGX Precast Protein Gels (4561086, Bio-Rad, Watford, UK) were loaded with 3 μg of protein per lane along with 4 μL of PageRuler Plus Prestained Protein Ladder, 10 to 250 kDa (26619, Thermo Fisher Scientific) and run at 120V in 1× Tris-Glycine-SDS Buffer (10x Tris/glycine/SDS, 1610772, Bio-Rad), followed by staining with InstantBlue (ISB1L, Expedeon, Cambridge, UK) to visualize protein bands. 

### 4.9. Binding Assay of Anti-CSPG4 IgG 

Binding of anti-CSPG4 IgG to A375, A2058, SBCL-2 and SKBR-3 cells was performed by using 10^5^ cells per sample. Cells were detached by using PBS plus 5 mmol/L EDTA. A 3 μM dilution of anti-CSPG4 IgG was prepared in FACS buffer (PBS and 2% FBS). Primary antibodies were incubated for 30 minutes at 4 °C, followed by a wash with 3 mL of FACS buffer (spinning at 500× *g* for 5 minutes). Samples were incubated with the secondary antibody goat anti-human IgG–fluorescein isothiocyanate (FI-3080; Vector Laboratories, Peterborough, UK) at 30 μg/mL in FACS buffer per sample for 30 minutes at 4 °C, followed by 1 wash as above. Samples were resuspended in 100 μL of FACS buffer and analyzed with a FACSCanto II (BD Biosciences, Wokingham, UK).

### 4.10. Cell Viability Assay

10^3^ cells were seeded per well in 96-well plates and treated with indicated amounts of antibody, ADC, payload and dummy payload. Cell viabilities were detected by methyl tetrazolium assay (G3580, Promega, Chilworth, UK). Optical absorbance was read on FLUOstar Omega spectrophotometer (BMG Labtech, Aylesbury, UK) to determine viable cell counts after 96 hours. 

### 4.11. Confocal Microscopy

For antibody internalization studies 2 × 10^4^ A375 melanoma cells were seeded on coverslips in 24-well plates and treated 20 µL of 100 nM anti-CSPG4 IgG1 antibody at 5 min to 4 hours either at 4 °C or 37 °C. Cells were then washed twice with PBS, fixed with 4% paraformaldehyde (PFA) in PBS for 10 min at room temperature, washed with PBS-T (0.1% Triton in PBS) and incubated for 1 hour at room temperature in blocking buffer (5% FBS in PBS-T). Cells were stained with anti-IgG-647 (Thermo Fisher Scientific, A-21445, 1:1000) in blocking buffer for 1 hour at room temperature, washed 3 times in PBS-T and mounted on slides using prolong Gold Antifade Reagent with DAPI (Thermo Fisher Scientific, P35931). Samples were imaged at the Point Scanning Confocal A1R microscope (Nikon Centre, King’s College London, London, UK). Intracellular and total fluorescence values were measured, and antibody internalization analysis was performed by calculating the percentage of internal fluorescence divided by total fluorescence using ImageJ.

For PDD localization studies, the coumarin-labelled PDD derivative was prepared using synthetic methodologies previously reported in the literature for pyrrolobenzodiazepines analogues. In the first stage the PDD core was synthesized based on previously established routes [52], and the fluorescent diethylamino-coumarin moiety was then attached using standard amine coupling methodology according to a previously published method by Thurston and co-workers [53]. For staining, 2 × 10^4^ SKBR-3 cancer cells were seeded in 24-well plates and treated with 500 µl of 100 µM PDD. After 3 hours, the cells were washed twice with PBS, fixed with 4% PFA in PBS for 10 min at room temperature. Then, the cells were washed with PBS and stained with Wheat Germ Agglutinin Alexa Fluor^TM^ 633 Conjugate (Thermo Fisher Scientific, W21404, 5 µg/mL), for 10 min and washed with PBS. The samples were imaged using the Inverted Spinning Disk Confocal microscope (Nikon Centre, King’s College London).

### 4.12. Cell Counting and Apoptosis assay

A375 and WM1361 cells were seeded at 2 × 10^5^ cells per well in a 6-well plate and treated with 100 µl of 100 nM anti-CSPG4-PDD and incubated for 96 hours. Supernatants and detached cells were washed once in 1× Binding Buffer (included in Annexin V Apoptosis Detection Kit APC, 88-8007-72, eBioscience) and resuspended in 2 mL. Then, 1 mL of the suspension was used per sample, centrifuged and 5 µL of APC-conjugated Annexin V were added to 100 μL of cell suspensions and cells were then incubated for 10–15 minutes at room temperature. Cells were washed in 1× Binding Buffer and resuspended in 200 µL of 1 x binding buffer. Cells were then treated with 100 μL of DAPI (D1360, Thermo Fisher Scientific) staining solution (1:10,000) and 10 µL of CountBright^TM^ Absolute Counting Beads (C36950, Invitrogen^TM^, Loughborough, UK) and immediately analyzed on a FACSCanto II^TM^ flow cytometer. Cells were then analyzed by gating early apoptosis (Annexin+, DAPI-), late apoptosis (Annexin+, DAPI+), necrosis (Annexin-, DAPI+) and as well as live cells (Annexin-, DAPI-).

### 4.13. In Vivo Procedures 

Animals were handled in accordance with Institutional Committees on Animal Welfare (The Home Office Animals Scientific Procedures Act, 1986). MTD: Six-week- old female athymic nude mice received single intravenous injection of 2.5, 5, 7.5 or 10 mg/kg anti-CSPG4-PDD and weight change was monitored every 3-4 days. Experiments were terminated after 18 days or if >15% weight loss was observed. 

Efficacy 2 mg/kg: Six-week- old female athymic nude mice were used for subcutaneous injection of 12. × 10^6^ A375 cells (in 100 μL PBS) (day 0). On day 10 and day 24, mice received single intravenous injection of 2 mg/kg anti-CSPG4-PDD, 2 mg/kg isotype-PDD, PDD (molar equivalent of 2 mg/kg ADC) or anti-CSPG4 + PDD (molar equivalent of 2 mg/kg ADC). Tumors were measured with calipers and volumes calculated (length × width^2^/ 2). Experiments were terminated after 40 days or when tumor sizes were ≤ 750 mm^3^. Efficacy 5 mg/kg: Six-week-old female athymic nude mice were used for subcutaneous injection of 12. × 10^6^ A375 cells (in 100 μL PBS) (day 0). On day 10, mice received single intravenous injection of 5 mg/kg anti-CSPG4-PDD or 5 mg/kg isotype-PDD. Tumors were measured with calipers and volumes calculated (length × width^2^/2). Experiments were terminated after 75 days or when tumor sizes were ≤ 750 mm^3^. On day 70, three tumors of anti-CSPG4-PDD treated mice and one tumor from an isotype control IgG1-PDD treated mouse were harvested, washed with PBS, the tissue was gently mashed, strained (0.2 μm) and cultured with penicillin-streptomycin for 2 weeks until cells were used for ex vivo cell viability assays. 

## 5. Conclusions

In summary, we present the first example of an ADC targeting the neural crest antigen CSPG4, that has potent anti-melanoma activity. This ADC delivers to tumor cells a highly cytotoxic pyrridinobenzodiazepine (PDD)-based payload that works by forming mono-alkylated DNA adducts within the DNA minor groove which, unlike the more widely known pyrrolobenzodiazepine (PBD) DNA cross-linking agents, are thought to have enhanced tolerability profiles. In vitro and in vivo efficacy and preliminary toxicity evaluations have highlighted the promise of this ADC for potential further development. The potency of this ADC, in the nanomolar to picomolar range, and favorable pre-clinical in vitro and in vivo toxicity profiles, as well as the retained susceptibility of residual melanoma cells to the ADC, are consistent with the reported DNA minor groove sequence-selective binding and tumor efflux-resistant nature of the PDD payload class [29]. Considering the unmet clinical need for new effective therapeutic agents to treat malignant melanoma, our findings point to this ADC approach as offering a potential new treatment mode for a large proportion of patients with aggressive melanotic disease. Further evaluations of this agent and its potential beneficial effects for melanoma patients who have failed on existing targeted and checkpoint inhibitor therapies are warranted. 

## Supplementary Materials

The following are available online at https://www.mdpi.com/2072-6694/12/4/1029/s1, Figure S1: Conjugation of Isotype-IgG1 antibody to PDD payload via stochastic conjugation generates Isotype-(PDD) ADC with a Drug Antibody Ratio (DAR) of 2.1, Figure S2: SDS-PAGE gel of anti-CSPG4-(PDD), Figure S3: MTD study for the CSPG4-(PDD) ADC in female athymic nude mice based on animal weight.
