# Supplementary material for: A Novel Antibody-Drug Conjugate (ADC) Delivering a DNA Mono-Alkylating Payload to Chondroitin Sulfate Proteoglycan (CSPG4)-Expressing Melanoma
Source: Cancers (Basel). 2020 Apr 22;12(4):1029. doi: 10.3390/cancers12041029 (PMC7226475; doi:10.3390/cancers12041029)
Supplement: Supplementary file 1 [file cancers-12-01029-s001.pdf]

Supplementary Materials: A Novel Antibody-Drug Conjugate (ADC) Delivering a DNA Monoalkylating Payload to Chondroitin Sulfate Proteoglycan (CSPG4)-Expressing Melanoma

Ricarda M. Hoffmann, Silvia Crescioli, Silvia Mele, Eirini Sachouli, Anthony Cheung, Connie K. Chui, Paolo Andriollo, Paul J. M. Jackson, Katie E. Lacy, James F. Spicer, David E. Thurston and Sophia N. Karagiannis

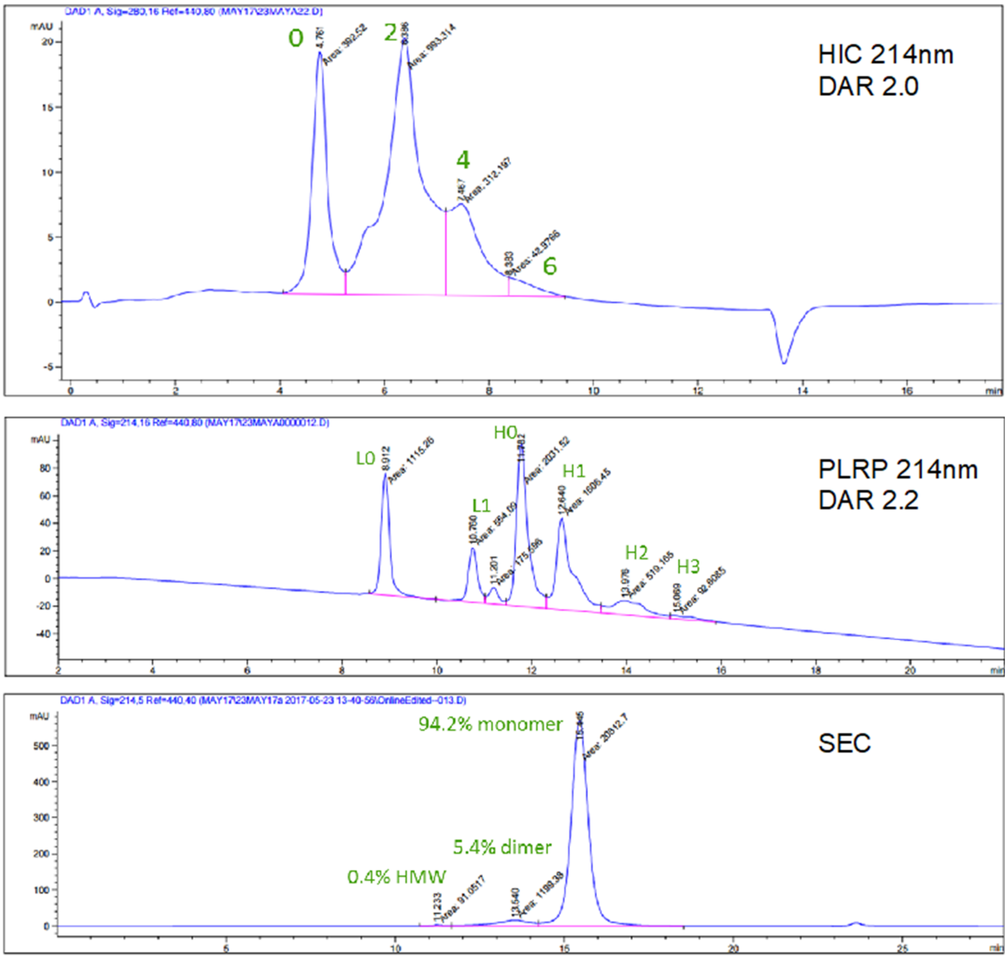

Figure S1. Conjugation of Isotype-IgG1 antibody to PDD payload via stochastic conjugation generates Isotype-(PDD) ADC with a Drug Antibody Ratio (DAR) of 2.1. Key analytical data from the stochastic conjugation of the isotype-IgG1 antibody to the PDD. The HIC (top panel) and PLRP (middle panel) analyses confirm an average Drug-Antibody Ratio (DAR) of 2.0 and 2.2 respectively, and the Size Exclusion Chromatography (SEC) trace (bottom panel) indicates negligible aggregation of the ADC and no detection of free linker-payload.

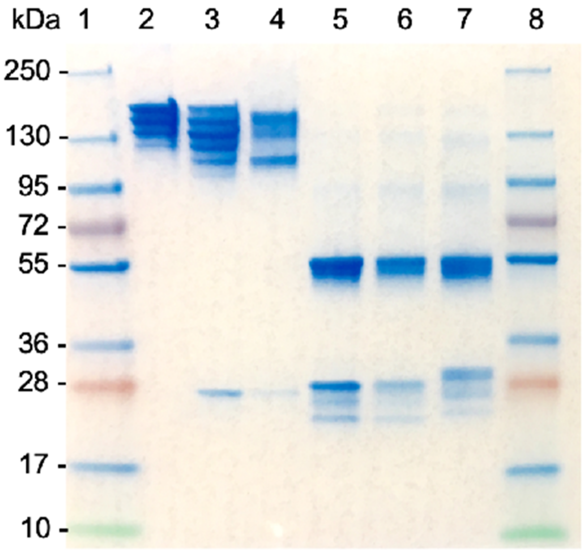

Figure S2. SDS-Page gel of anti-CSPG4-(PDD). SDS-Page followed by InstantBlue™ staining 1& 8: Molecular weight standard; 2: anti-CSPG4 antibody; 3: anti-CSPG4-(PDD); 4: isotype-(PDD); 5: anti-CSPG4 antibody reduced; 6: anti-CSPG4-(PDD) reduced; 7: isotype-(PDD) reduced.

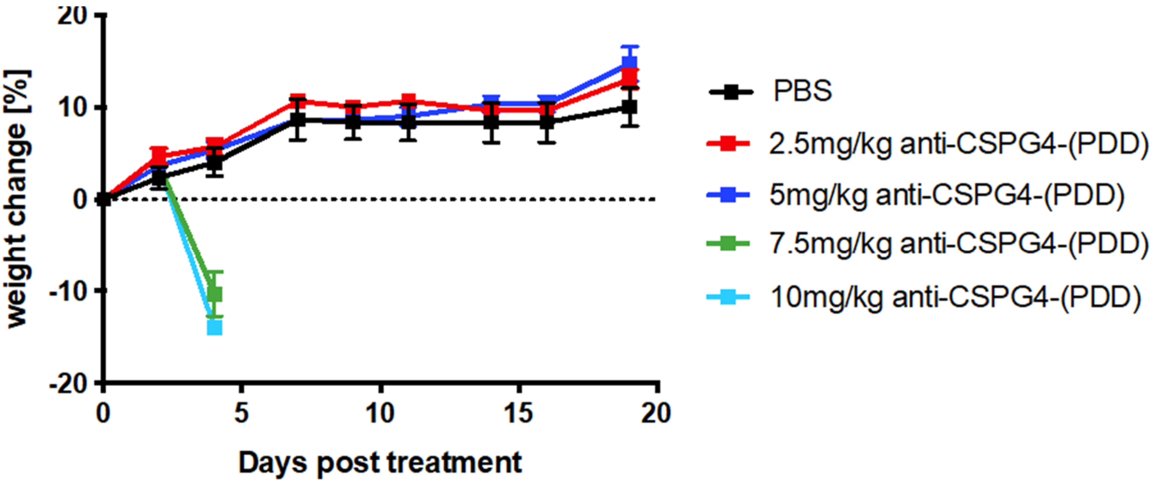

Figure S3. MTD study for the CSPG4-(PDD) ADC in female athymic nude mice based on animal weight. Non-tumour bearing animals were injected (*i.v.*) with a single dose of anti-CSPG4-(PDD) (2.5–10 mg/kg). Apart from weight loss at the higher doses, no other symptoms of toxicity were observed ($n = 3$ mice per group, error bars represent SEM).

© 2020 by the authors. Submitted for possible open access publication under the terms and conditions of the Creative Commons Attribution (CC BY) license (http://creativecommons.org/licenses/by/4.0/).
